# Research topic: neuromorphic engineering systems and applications. A snapshot of neuromorphic systems engineering

**DOI:** 10.3389/fnins.2014.00424

**Published:** 2014-12-19

**Authors:** Tobi Delbruck, André van Schaik, Jennifer Hasler

**Affiliations:** ^1^Institute of Neuroinformatics, University of Zurich and ETH ZurichZurich, Switzerland; ^2^Bioelectronics and Neuroscience, The MARCS Institute, University of Western SydneySydney, NSW, Australia; ^3^School of Electrical and Computer Engineering, Georgia Institute of TechnologyAtlanta, GA, USA

**Keywords:** neuromorphic engineering, neural networks, event-based, spiking neural networks, dynamic vision sensor, floating gate, neural simulation, synaptic plasticity

The 14 papers in this research topic were solicited primarily from attendees to the two most important hands-on workshops in neuromorphic engineering: the *Telluride Neuromorphic Cognition Engineering Workshop* (www.ine-web.org) and the *Capo Caccia Cognitive Neuromorphic Engineering Workshop* (capocaccia.ethz.ch). The papers show the results of feasibility studies of new concepts, as well as neuromorphic systems that have been constructed from more established neuromorphic technologies. Five papers exploit neuromorphic dynamic vision sensor (DVS) events that mimic the asynchronous and sparse spikes on biology's optic nerve fiber (Delbruck and Lang, 2013; O'Connor et al., 2013; Rea et al., 2013; Brandli et al., 2014; Camunas-Mesa et al., 2014; Clady et al., 2014). Two papers are on the hot topic (based on largest number of views) of event-driven computation in deep belief networks (DBNs) (O'Connor et al., 2013; Neftci et al., 2014). Two papers use floating gate technology for neuromorphic analog circuits (Gupta and Markan, 2014; Marr and Hasler, 2014). The collection is rounded out by papers on central pattern generators (Ambroise et al., 2013), neural fields for cognitive architectures (Sandamirskaya, 2014), sound perception (Coath et al., 2014), polychronous spiking networks (Wang et al., 2014), and automatic parameter tuning for large network simulations (Carlson et al., 2014).

## Regarding the event-based vision papers

Brandli et al. (2014) report on a novel method for rapidly and cheaply tracking a flashing laser line using a DVS, which is aimed at building a fast obstacle detector for small ground-based robots, such as vacuum cleaners or toy cars. Particular novelties in this paper are the adaptive temporal filter and the efficient algorithmic update of the laser line position.

Delbruck and Lang (2013) report on the detailed implementation of a fun robotic goalie, which uses a DVS to help track the balls and robot arm. The paper includes measurements of USB latency. The novelty in this paper is the self-calibration of the goalie arm so that it can be rapidly placed in a particular place in visual space. Both of the preceding two papers include YouTube citations to videos and both have open-source implementations.

Rea et al. (2013) report on the integration of a stereo pair of DVS sensors with the iCub robot, and how they are used for quick, low power saliency detection for the iCub. In particular, the Koch-Ittie visual saliency model was adapted to event-driven sensors and many experiments were done to characterize its effectiveness and efficiency.

Camunas-Mesa et al. (2014) reports on a stereo vision system that uses a pair of their DVS cameras together with an FPGA that computes low level oriented features. This paper has a wealth of characterization results.

Clady et al. (2014) report the first results of computing the old problem of time to contact (TTC) for a moving entity from DVS events. They take a geometrical approach in order to extract low level motion features from the DVS events to obtain the TTC information. This paper includes robotic experiments.

## Regarding event-driven deep networks

O'Connor et al. (2013) present the first of the papers to focus on event-based learning and networks. Their system also uses a DVS, as well as an AEREAR2 binaural silicon cochlea, to build a spike-based DBN for recognizing MNIST digits presented in conjunction with pure tones. They demonstrate that a DBN constructed from stacks of restricted Boltzmann machines (RBMs) is valuable for learning and computing sensor fusion. They also show that a DBN's recurrent persistent activity is useful particularly with sparse event-driven sensor input. This network was trained off-line, and then the weights were transferred onto the spiking network.

Neftci et al. (2014) report on the same target application of MNIST digit recognition, but their paper takes a further step by proposing how a network of integrate and fire neurons can implement a RBM, and can be trained with an event-driven version of the well-known contrastive divergence training algorithm for RBMs.

## Regarding floating gate technology

Two papers show the versatility of floating-gate (FG) circuit approaches. Marr and Hasler (2014) describe a collaborative project started and effectively completed during the Telluride 2008 workshop as a representative of the possible opportunity at any of these workshops. In this case, the opportunity was enabled through the use of large-scale field programmable analog arrays (FPAA) as a mixed mode processor for which functions can be compiled enabling a range of circuit, system, and application design. The focus was on stochastic computations that are dynamically controllable via voltage-controlled amplifiers and comparator thresholds. From Bernoulli variables it is shown that exponentially distributed random variables, and random variables of an arbitrary distribution can be computed. The trajectory of a biological system computed stochastically with this probabilistic hardware results in a 127X performance improvement over current software approaches.

Gupta and Markan (2014), report on a FG adaptive system for investigating self-organization of image patterns. They describe adaptive feature selectivity as a mechanism by which nature optimizes resources so as to have greater acuity for more abundant features. The authors look to exploit hardware dynamics to build adaptive systems utilizing time-staggered winner-take-all circuits, exploiting the adaptation dynamics of FG transistors, to model an adaptive cortical cell.

## Regarding other topics in network architectures

Wang et al. (2014) report results from a polychronous multineuron chip. Polychronization is the process in which spikes travel down axons with specific delays to arrive at a common target neuron simultaneously and cause it to fire, despite the source neurons firing asynchronously. This paper shows digital and analog tradeoffs and offers advice for scaling to future technologies.

Ambroise et al. (2013) describe a neuromorphic implementation of a network of 240 Central Pattern Generator modules modeling the leech heartbeat neural network on a field programmable gate array. It uses the Izhikevich neuron model, implemented as a single computational core, time multiplexed to update all the neurons in the network. In order to fit the digital implementation to the data from the biological system without implementing all the detailed synaptic dynamics, which would take up too many resources, they propose a new synaptic adaptation model: an activity-dependent depression synapse.

Sandamirskaya (2014) leverages the relationship between dynamic field theory networks and neuromorphic circuits using soft winner take all circuits (WTA) to formally describe the equivalence between the two and establish a common ground. It sets a possible roadmap for the development of cognitive neuromorphic systems using WTA implementations.

Coath et al. (2014) describes a pattern recognition network implemented using a column of three neurons in which the columns are connected via axons with delays that explicitly depend on the distance between the columns. The networked is trained using spike-timing dependent plasticity and it is shown that the performance of the network is robust to natural variations in the input stimuli.

Carlson et al. (2014) address the significant problem of finding solutions in the enormous parameter space found in implementations of spiking neural networks by proposing an automated tuning framework. Their approach uses evolutionary algorithms implemented on graphics processing units for speed. They use an objective function based on the Efficient Coding Hypothesis to tune these networks. In their example, they demonstrate the evolution of V1 simple cell responses. Using GPU parallelization, they report 65x speedups over CPU implementations.

## Summary

Amidst the promises offered by projects with major chunks of funding in neuromorphic engineering like HBP, BrainScaleS, SpiNNaker, and TrueNorth, this research topic offers a refreshing glimpse into some of the current actual accomplishments in neuromorphic systems engineering and applications.

### Conflict of interest statement

The Associate Editor Giacomo Indiveri declares that, despite being affiliated to the same institution as author Tobi Delbruck, the review process was handled objectively and no conflict of interest exists. The authors declare that the research was conducted in the absence of any commercial or financial relationships that could be construed as a potential conflict of interest.

## References

[B1] Ambroise M. Levi T. Joucla S. Yvert B. Saïghi S. (2013). Real-time biomimetic central pattern generators in an FPGA for hybrid experiments. Front. Neurosci. 7:215. 10.3389/fnins.2013.00215 24319408 PMC3836270

[B2] Brandli C. Mantel T. A. Hutter M. Delbruck T. (2014). Adaptive pulsed laser line extraction for terrain reconstruction using a dynamic vision sensor. Front. Neurosci. 7:275. 10.3389/fnins.2013.00275 24478619 PMC3894568

[B3] Camunas-Mesa L. A. Serrano-Gotarredona T. Ieng S. H. Benosman R. B. Linares-Barranco B. (2014). On the use of orientation filters for 3D reconstruction in event-driven stereo vision. Front. Neurosci. 8:48. 10.3389/fnins.2014.00048 24744694 PMC3978326

[B4] Carlson K. D. Nageswaran J. M. Dutt N. Krichmar J. L. (2014). An efficient automated parameter tuning framework for spiking neural networks. Front. Neurosci. 8:10. 10.3389/fnins.2014.00010 24550771 PMC3912986

[B5] Clady X. Clercq C. Ieng S.-H. Houseini F. Randazzo M. Natale L.. (2014). Asynchronous visual event-based time-to-contact. Front. Neurosci. 8:9. 10.3389/fnins.2014.00009 24570652 PMC3916774

[B6] Coath M. Sheik S. Chicca E. Indiveri G. Denham S. Wennekers T. (2014). A robust sound perception model suitable for neuromorphic implementation. Front. Neurosci. 7:278. 10.3389/fnins.2013.00278 24478621 PMC3894459

[B7] Delbruck T. Lang M. (2013). Robotic goalie with 3ms reaction time at 4% CPU load using event-based dynamic vision sensor. Front. Neurosci. 7:223. 10.3389/fnins.2013.00223 24311999 PMC3836084

[B8] Gupta P. Markan C. M. (2014). An adaptable neuromorphic model of orientation selectivity based on floating gate dynamics. Front. Neurosci. 8:54. 10.3389/fnins.2014.00054 24765062 PMC3980111

[B9] Marr B. Hasler J. (2014). Compiling probabilistic, bio-inspired circuits on a field programmable analog array. Front. Neurosci. 8:86. 10.3389/fnins.2014.00086 24847199 PMC4019887

[B10] Neftci E. Das S. Pedroni B. Kreutz-Delgado K. Cauwenberghs G. (2014). Event-driven contrastive divergence for spiking neuromorphic systems. Front. Neurosci. 7:272. 10.3389/fnins.2013.00272 24574952 PMC3922083

[B11] O'Connor P. Neil D. Liu S.-C. Delbruck T. Pfeiffer M. (2013). Real-time classification and sensor fusion with a spiking deep belief network. Front. Neurosci. 7:178. 10.3389/fnins.2013.00178 24115919 PMC3792559

[B12] Rea F. Metta G. Bartolozzi C. (2013). Event-driven visual attention for the humanoid robot iCub. Front. Neurosci. 7:234. 10.3389/fnins.2013.00234 24379753 PMC3862023

[B13] Sandamirskaya Y. (2014). Dynamic neural fields as a step toward cognitive neuromorphic architectures. Front. Neurosci. 7:276. 10.3389/fnins.2013.00276 24478620 PMC3898057

[B14] Wang R. M. Hamilton T. J. Tapson J. van Schaik A. (2014). A mixed-signal implementation of a polychronous spiking neural network with delay adaptation. Front. Neurosci. 8:51. 10.3389/fnins.2014.00051 24672422 PMC3957211

